# Effect of adjuvants on physicochemical properties of lime sulfur on flower/paraffin and application on flower thinning

**DOI:** 10.3389/fpls.2023.1257672

**Published:** 2023-09-11

**Authors:** Yuanyuan Li, Yang Liu, Changjie Wu, Rui Zhao, Minghua Li, Jing Cai, Li Ma, Xiongkui He, Xuemin Wu, Zhang Zhenhua

**Affiliations:** ^1^ College of Science, China Agricultural University, Beijing, China; ^2^ College of Plant Protection, China Agricultural University, Beijing, China; ^3^ Qingshengyuan Agricultural Development Co., Ltd., Chengde, Hebei, China

**Keywords:** tank-mixture adjuvant, lime sulfur, flower thinning, dioctyl sulfosuccinate sodium salt, apple cultivation, sustainable agriculture

## Abstract

**Introduction:**

Adjuvants can effectively enhance the utilization rate of pesticides, but the application of adjuvants in plant growth regulators is rarely studied.

**Methods:**

This work explored the effects of adjuvants dioctyl sulfosuccinate sodium salt (AOT) and methyl oleate (MO) on lime sulfur (LS), especially the drop behavior on flower and paraffin surface.

**Results:**

The results showed that the addition of AOT and AOT+MO can significantly reduce the static and dynamic surface tension of LS from 72mN/m to 28mN/m and 32mN/m respectively, and increase the spreading factor from 0.18 to 1.83 and 3.10 respectively, reduce the bounce factor from 2.72 to 0.37 and 0.27 respectively. The fluorescence tracer test showed that the addition of adjuvants could promote the spreading and permeation of droplets. The field test results revealed that the flower thinning rate of adjuvant and non-adjuvant were 80.55% and 54.4% respectively, and the flower thinning effect of adding adjuvant was the same as that of artificial which the flower thinning rate was 84.77%. The quality of apples treated with adjuvants was similar to that treated with artificial, and the weight of single fruit increased by 24.08% compared with CK (spray water).

**Discussion:**

The application of tank-mixture adjuvant could reduce the dosage of LS for thinning agent application, improve apple’s quality, and decrease labor cost and improve the economic benefits of fruit planting and the environmental benefits of plant growth regulators.

## 1 Introduction

Flower and fruit thinning is an important technology in apple cultivation, which can reduce the number of fruits per plant to improve the quality of apples and promote the growth of apple trees (Ngugi and Schupp, 2009). At present, manual, mechanical, and chemical thinning strategies are commonly used for fruit thinning. However, mechanical thinning can damage flower and leaf, reduce photosynthesis, and in some cases, facilitate the spreading of fire blight in apple orchards. Manual thinning is an expensive, labor-intensive form of field management. With an aging population, skilled labor for thinning is not easy to be found in China.

Chemical thinning for flowers and fruits is conducted as it can save time and labor, and can achieve desired thinning in a time-effective manner. When metamitron, a chemical fruit thinning agent, was applied to fruit trees, fruit number per plant reduced, average fruit color improved, and fruit weight per plant and diameter significantly increased by thinning (Gonzalez et al., 2020). The thinning efficiency of metamitron was found to strongly correlate with night temperature. Lucas De Ross Marchioretto et al. have reported that spraying ammonium thiosulfate (ATS) affects the germination of pollen and achieves flower thinning (Marchioretto et al., 2019). Lime sulfur (LS) mixture treatment can not only control fungi, bacteria and insects, but also inhibit the growth of pollen tubes (Holb et al., 2003; Marchioretto et al., 2019). Mineral oil and ATS can achieve flower thinning under field conditions (Marchioretto et al., 2019). Growth regulators, such as 6-benzylaminopurine hydrochloride (6-BA), gibberellic acid (GA_4 + 7_) + 6-BA, 1-naphthaleneacetic acid (NAA), can significantly reduce crop load and improve fruit quality (Marchioretto et al., 2019). In these chemical thinnings of fruit and flower, the effect needs to be accurate and predictable, and the chemical agents needs to be reduced to have a wide window of concentration for safe usage (Lordan et al., 2018).

The addition of adjuvants can improve the wetting behavior of pesticide droplets, increase deposition of liquid on the target, facilitate the infiltration and transfer of active ingredients (Kovalchuk et al., 2014; Grundke et al., 2015). Oil adjuvants, mainly including mineral oil, vegetable oil, and vegetable oil derivatives, can promote the diffusion, adhesion, infiltration, and absorption of pesticide drops on leaves (Buchholz, 2006; Arand et al., 2010; Xu et al., 2011). As mineral oil is harmful, it should be sparingly used (Meng et al., 2016). Surfactants can significantly inhibit the fragmentation and rebound behavior of droplets impacting the leaf surface of hydrophobic plants and can improve the wetting and spreading behavior of droplets. For example, Wu, Zhang, Xu and coworkers reported that the use of appropriate tank-mix adjuvants at low dilution ratios for UAV application in paddy fields could improve the performance of spray dilutions, increase the effective deposition and wetting spread of pesticides on rice leaves, and further reduce the dosage of pesticide products and improve pesticide utilization (Zhao et al., 2022). In recent years, double-chain ionic surfactants, which have superspreading and superwetting effects, such as dioctyl sulfosuccinate sodium salt (AOT) and didecyldimethylammonium bromide (DDAB), have attracted great attention in pesticide application (Song et al., 2017; Song et al., 2019; Li et al., 2021). Jiang, Wang, Dong and coworkers showed that binary additive (0.005% PEO and 0.1% AOT) droplet have excellent spreading performance on superhydrophobic leaves, including rice, cauliflower, chive and cabbage (Song et al., 2019). Du, Gao and coworkers reported that DDAB can not only inhibit droplet regression and rebound but also significantly improve the herbicide control effect as observed through field experiments (Li et al., 2021). However, only few studies are available on the synergistic effects of adjuvants in plant growth regulation, and the study their behaviors on flower and paraffin surface are also rare.

Herein, we aimed to study the tank-mixture of AOT and methyl oleate (MO) into LS thinning agent to improve droplet performance on flower and paraffin, to achieve efficient, accurate, and appropriate flower thinning, to reduce the use of flower thinning agents, and to improve the efficacy on apple cultivation. We systematically studied the physicochemical properties of AOT and AOT + MO, their combination with LS. The impact behavior of different droplets on the target lowers and paraffins, and the spread and penetration characteristics of different droplets assessed using the fluorescent tracer method were estimated. The addition of adjuvants can reduce the dosage of LS thinning agent and the effect is as good as that of thinning by artificial. As a result, the dosage of thinning agent application was reduced, the apple’s quality was improved, and labor cost was further decreased and improve the economic benefits of fruit planting and the environmental benefits of plant growth regulators.

## 2 Materials and methods

### 2.1 Materials

Lime sulfur (LS) comprised of calcium oxide, sulfur and water in a ratio of 1:2:10 was made at Qingshengyuan Agricultural Development Co., Ltd. (China). The adjuvants, namely, bis(2-ethylhexyl) sodium sulfosuccinate (AOT, 97%), methyl oleate (MO), and emulsifier were purchased from Aladdin Co., Ltd. (China), Hebei Ming Shun Agricultural Science and Technology Co., Ltd. (China) and Nantong deyi Chemical Co., Ltd. (China) respectively. 1,3,6-Pyrenetrisulfonicacid,8-hydroxy-trisodiumsalt (pyranine) was purchased from Shanghai Maclin Biochemical Technology Co., Ltd. (China). An AOT + MO mixture of AOT (35%) + MO (55%) + emulsifier (10%) was prepared at China Agricultural University. Flat paraffin plates were prepared by melting solid paraffin, using it to cover the slide, and letting it cool to room temperature.

### 2.2 Static surface tension

Static surface tension was measured using the Wilhelmy plate method using an automatic tension meter, JK99B (Shanghai Zhong Chen Digital Technology Equipment Co., Ltd.). The adjuvant solutions were diluted in distilled water, and the critical micellar concentration curve was plotted by taking the average of three measurements.

### 2.3 Wetting experiments

Contact angles were measured using the sessile drop method using an OCA 15 Plus optical contact angle measuring device (Data Physics Instruments GmbH, Filderstadt, Germany). Each treatment was repeated 5 times. Recording was performed at a speed of 0.45 fps, and the shooting process lasted for 5 min.

### 2.4 Adhesion work

The adhesion work (Wa) of the solutions can be calculated using Eq. (1) (Lee and Lee, 2011):


(1) Wa =γSV +γLV −γSL 

The Young"s equation is expressed as Eq. (2):


(2) γSV −γSL =γLV cosΦ 

Substituting Eq. (1) into Eq. (2), we obtain:


(3) Wa =γLV (1 + cosΦ) 

According to Eq. (3), by measuring the contact angle and surface tension of pesticide solution on the surface of paraffin and petals, the adhesion work can be calculated (Zheng et al., 2021).

### 2.5 Impact experiments

Impact experiments were performed by high-speed photography method using a camera (I - Speed 220, IX - cameras, UK). The impact progress was recorded at 4021 fps and 592 × 534 px from 0° and 30° views. The droplets fell from a peristaltic pump (LD - P2020II, Shanghai Lande Medical Equipment Co. Ltd.) on to the surface of a flat paraffin plate. Droplets were generated using flat-tipped syringe needles with internal diameters of 0.17 or 0.6 mm. Plant targets were of the same size and a constant impact velocity was maintained. The droplets had a diameter of approximately 2 ± 0.2 mm, and they fell on the surface at an impact velocity of 2 m/s. The images were analyzed using ImageJ to quantitatively track the droplet’s impact process.

### 2.6 Dynamic surface tension

Dynamic surface tension was measured using the maximum bubble pressure method using the bubble pressure tensiometer BPA - 2P (SINTERFACT, Germany). The tendency of surface tension within 10 ms to 10 s was measured to characterize the dynamics of adsorption of surface-active compounds.

### 2.7 Fluorescent tracer experiments

The spreading and penetrating properties of droplets on plant targets were measured using fluorescent tracer method using 1% pyranine, a fluorescent dye, and ultraviolet light. Pyranine was applied on the stamens, pistils, and petals of apple flowers. After 12 h without dew and rain, the apple flowers were removed for indoor photography experiments to observe the behavior of pyranine, excited using a handheld 365 nm ultraviolet lamp.

### 2.8 Field Experiments

#### 2.8.1 Field experimental design and treatment

The chemical thinning experiments on apple cultivation were performed at Qingshengyuan Agricultural Development Co. LTD., Pingquan city, Hebei Province, from May 1 to October 20, 2021. The Yueguan variety, an experimental variety (hybrid of Hanfu and Yueshuai varieties), was used. The following solutions were sprayed at a volume of 2 L/tree: LS at 0.5, 0.75, and 1 B° and 0.75 B° LS + 0.1% AOT and 0.75 B° LS + 0.1% AOT + 0.16% MO. Water, 0.1% AOT, 0.1% AOT + 0.16% MO, and artificially thinning were set as the controls in May 2021. Each treatment was performed on 2 trees; therefore, the experiment involved a total of 18 trees. Fruit trees with approximately the same perimeter (25-28 cm) of trunks, crown width(1.8-2 m × 2.2-2.5 m), and tree growth were selected before the experiment.

#### 2.8.2 Field experimental indices and determination methods

##### 2.8.2.1 Flower number and inflorescence number

Two repetitions were set per process, and on each tree two branches for four directions (north, east, south, and west) were marked. The fruit number and branch growth were similar among the trees. The flower and inflorescence numbers in each group of branches were counted before spraying the solutions.

##### 2.8.2.2 Fruit setting rate

In June, the rates of inflorescence fruit-set, total flower fruit-set, single/double fruit-set, single fruit-set, empty fruit, and flower thinning were calculated from the marked branches in each treatment group as follows.


inflorescence fruit set rate (%)= inflorescence fruit set number/total inflorescence number×100%

total flower fruit set rate (%) = number of fruits on inflorescence/number of flowers on inflorescence×100%

single/double fruit set rate (%) = inflorescence of single or double fruits set number/total inflorescence fruits set number×100%

single fruit set rate (%) = inflorescence of single fruits set number/total inflorescence fruits set number×100%

empty fruit rate (%) = inflorescence fruits not set number/total inflorescence fruits set number×100%

flower thinning rate (%) = thinning flowers number on inflorescence/number of flowers on inflorescence×100%

##### 2.8.2.3 Determination of fruit quality

After apple fruits were mature, 10 apples were randomly and evenly picked from the upper, middle, and lower levels of each tree in each treatment group. Therefore, 20 apples were selected from each treatment group, and their individual weight, hardness, soluble solid content, and vertical and horizontal diameters were measured. Fruit hardness was measured using GY - 3 fruit hardness tester; soluble solids were measured using BM - 0532 digital refractometer-saccharometer.

##### 2.8.2.4 Economic valuation

The amount of sprayed solution and expenditure were calculated in terms of hectares, and the ratio of chemical thinning cost to artificial thinning cost was calculated with artificial thinning as the denominator and each treatment as the numerator.

### 2.9 Statistical analyses

Data analysis involves taking the average of all duplicate values in the processing group. The obtained data were processed and analyzed using SPSS Statistics software (version 20.0), Origin (version 2021), and Excel data processing software. The fruit quality index was expressed as mean ± standard deviation.

## 3 Results and discussion

### 3.1 Critical micellar concentration curves of adjuvants

The critical micellar concentration is determined by the minimum concentration of surfactant molecules required to form micelles in solution. When the solution has critical micellar concentration, the surface tension of the solution decreases to the minimum value. At this time, even when the surfactant concentration is further increased, the surface tension of the solution is no longer reduced, but more micelles are formed.

**Figure 1:**
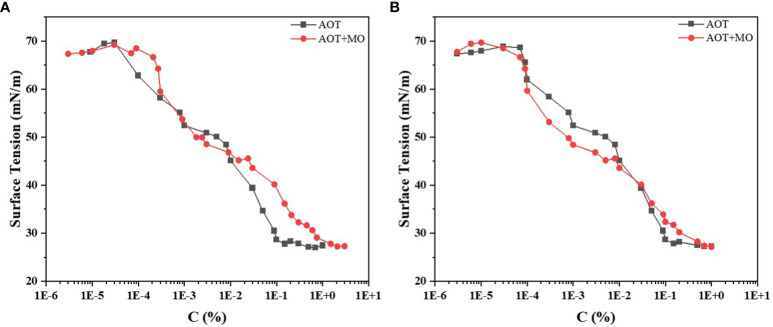
Critical micellar concentration curves of AOT and AOT + MO. **(A)** Critical micellar concentration curves of the two adjuvants, AOT and AOT + MO. **(B)** Critical micellar concentration curves of the two adjuvants at the same AOT concentration. AOT: dioctyl sulfosuccinate sodium salt MO: methyl oleate.

We measured the critical micellar concentration of (AOT) and AOT+ MO and plotted the critical micellar concentration curve (**Figures 1A, B**). At identical adjuvant concentrations (**Figure 1A**), AOT reached the inflection point earlier than AOT + MO. The critical micellar concentration of AOT was 0.1%, and that of AOT + MO was approximately 1%. The surface tension corresponding to the critical micellar concentration was approximately 27 mN/m.

The critical micellar concentration curve when the AOT concentration of the two adjuvants was the same is shown in **Figure 1B**. When the critical micellar concentration was less than 0.01%, the surface tension of AOT + MO at the same AOT concentration was smaller than that of AOT. However, when the critical micellar concentration was greater than 0.01%, it was the opposite. The concentration of AOT used in the experiment was 0.1% and the concentrations of AOT/MO were 0.1%/0.16%, respectively. The surface tensions of the two were 28 and 32 mN/m, respectively.

### 3.2 Wetting and spreading of the droplets

**Figure 2:**
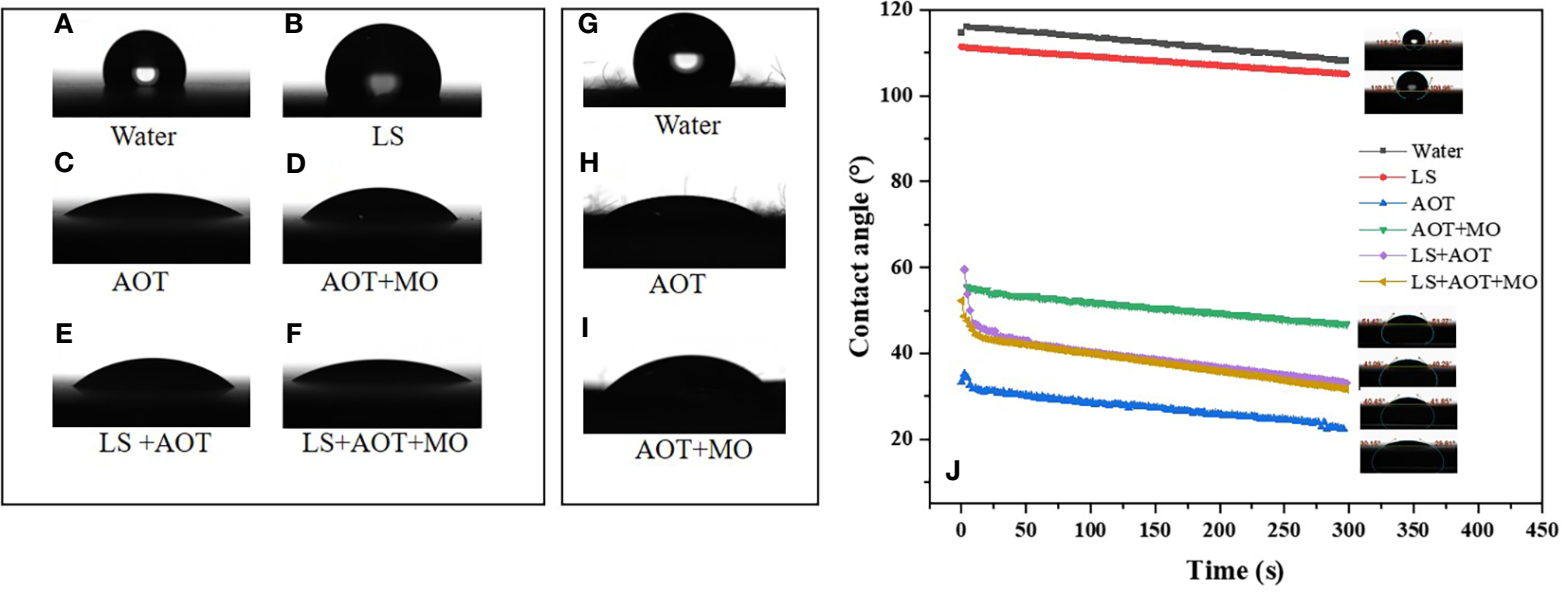
Contact angle of droplets on flat paraffin plate and petal surfaces. **(A–F)** The wetting and spreading of droplets on paraffin surfaces. **(A)** water, **(B)** 0.75 B° LS, **(C)** 0.1% AOT, **(D)** 0.1% AOT + 0.16% MO, **(E)** 0.75 B° LS + 0.1% AOT, and **(F)** 0.75 B° LS + 0.1% AOT + 0.16% MO. **(G–I)** The wetting and spreading of droplets on petal surfaces. **(G)** water, **(H)** 0.1% AOT, and **(I)** 0.1% AOT + 0.16% MO **(J)** The tendency of contact angles of the solutions on paraffin surfaces at 5 min. The treatments were water, 0.75 B° LS, 0.1% AOT, 0.1% AOT + 0.16% MO, 0.75 B° LS + 0.1% AOT, and 0.75 B° LS + 0.1% AOT + 0.16% MO. AOT, dioctyl sulfosuccinate sodium salt; MO, methyl oleate; LS, lime sulfur.

When a droplet touches a solid surface, a three-phase contact line is formed. When the droplet three-phase contact line stops moving, the droplet reaches the optimal wetting state (He et al., 2021). Wenzel model, Cassie-Baxter model, and Wenzel model and Cassie-Baxter transition state models are suitable for simulating the wettability of rough hydrophobic solid surfaces because of the presence of micro-nano structures (Quéré, 2003; Bormashenko, 2015). We evaluated the contact states of different droplets with and without adjuvants on flat paraffin plates and on petals of apple flowers, and observed the wetting states of different droplets (**Figure 2**).

On the flat paraffin plate, the wetting state of water and lime sulfur (LS) (0.75 B°) was close to the Cassie-Baxter model. However, after the addition of adjuvants, the droplets exhibited Wenzel and Cassie-Baxter transition state models, and the transition from Cassie-Baxter state to Wenzel state occurred. The contact angle of water on the petal surface (122°) is shown in **Figure 2G**. Petals, one of the targets of the flower thinning agent, have a hydrophobic surface. After adding AOT and AOT + MO, the state of the petal changed from Cassie-Baxter to Wenzel state. Particularly after adding AOT, the wetting state of the droplet was close to the Wenzel state. In the Cassie-Baxter state, the friction between the droplet and solid surfaces decreases, and the rolling angle becomes smaller, which makes it easier to roll off. In the Wenzel state, the friction between the droplet and solid surfaces increases, and the rolling angle becomes larger, which makes it easier to deposit (Quéré, 2003).

The contact angles of water and LS on the surface of paraffin were 116° and 108°, respectively (**Figure 2J**). However, after adding adjuvants, the contact angles of droplets were significantly reduced. The contact angles of AOT and AOT + MO on the surface of paraffin were approximately 30° and 51°, respectively. The contact angle of AOT with LS increased slightly compared with AOT alone; however, only a small difference was observed in the contact angle between the two adjuvants and LS mixtures. We hypothesize that the emulsifier in AOT + MO emulsifies the agent to reduce the droplet contact angle.

### 3.3 Mechanism of wettability of droplets on paraffin and petal surfaces

It is important to understand the interaction of pesticide droplets with plant surfaces. Target wettability largely determines the retention of pesticide droplets on the surface of crops and target plants (Armstrong et al., 2020; Gao et al., 2020; Zheng et al., 2021).

The wetting behavior of pesticide droplets on paraffin and petal surfaces were studied using adhesion work. The principal mechanism of the effect of surface tension and contact angle on wetting behavior is discussed below.

As shown in **Table 1**, the adhesion work of different droplets on the surface of paraffin surface was as follows. The adhesion work of water and pesticide droplets was slightly smaller, approximately 41 mJ/m^2^, and the adhesion work of droplets increased with the addition of the two adjuvants (approximately 54 mJ/m^2^). Compared with that of LS, the adhesion work of the LS and adjuvant combinations increased, particularly that of LS + AOT + MO, where the adhesion work was approximately higher by 10 mJ/m^2^ than LS. The higher the work of adhesion, the more the liquid can wet the solid (He et al., 2021).

**Table 1:** Adhesion work (Wa) of different droplets on paraffin.

Treatment	Surface Tension (mN/m)	Contact Angle (°)	Adhesion Work (mJ/m^2^)
Water	72.00	116.00	40.50
0.75 B° LS	59.89	108.12	41.31
0.1% AOT	28.63	28.11	53.88
0.1% AOT + 0.16% MO	32.26	47.55	54.05
0.75 B° LS + 0.1% AOT	25.31	35.96	45.80
0.75 B° LS + 0.1% AOT + 0.16% MO	28.23	37.58	50.61

AOT, dioctyl sulfosuccinate sodium salt; MO, methyl oleate; LS, lime sulfur; B°, baume degrees.

Additionally, the adhesion on petal surface exhibited the same rule as that on paraffin surface. As previously mentioned, the contact angle on the surface of apple flower petals was greater. Furthermore, the larger adhesion function is conducive to the deposition and adhesion of pesticide droplets on the surface of the target petals (**Table 2**), thus, reducing the splash and bounce of pesticide.

**Table 2:** Adhesion work (Wa) of different droplets on petal surfaces.

Treatment	Surface tension (mN/m)	Contact Angle(°)	Adhesion Work (mJ/m2)
Water	72.00	122.71	33.16
0.75 B°LS	59.89	115.43	34.22
0.1%AOT	28.63	36.18	51.74
0.3% (AOT+MO)	32.26	43.89	55.52
0.75 B°LS+0.1% AOT	25.31	42.16	44.07
0.75 B°LS + 0.1% AOT+0.16% MO	28.23	48.42	46.98

AOT, dioctyl sulfosuccinate sodium salt; MO, methyl oleate; LS, lime sulfur; B°, baume degrees.

### 3.4 Impact behavior of droplets on a flat paraffin plate

**Figure 3:**
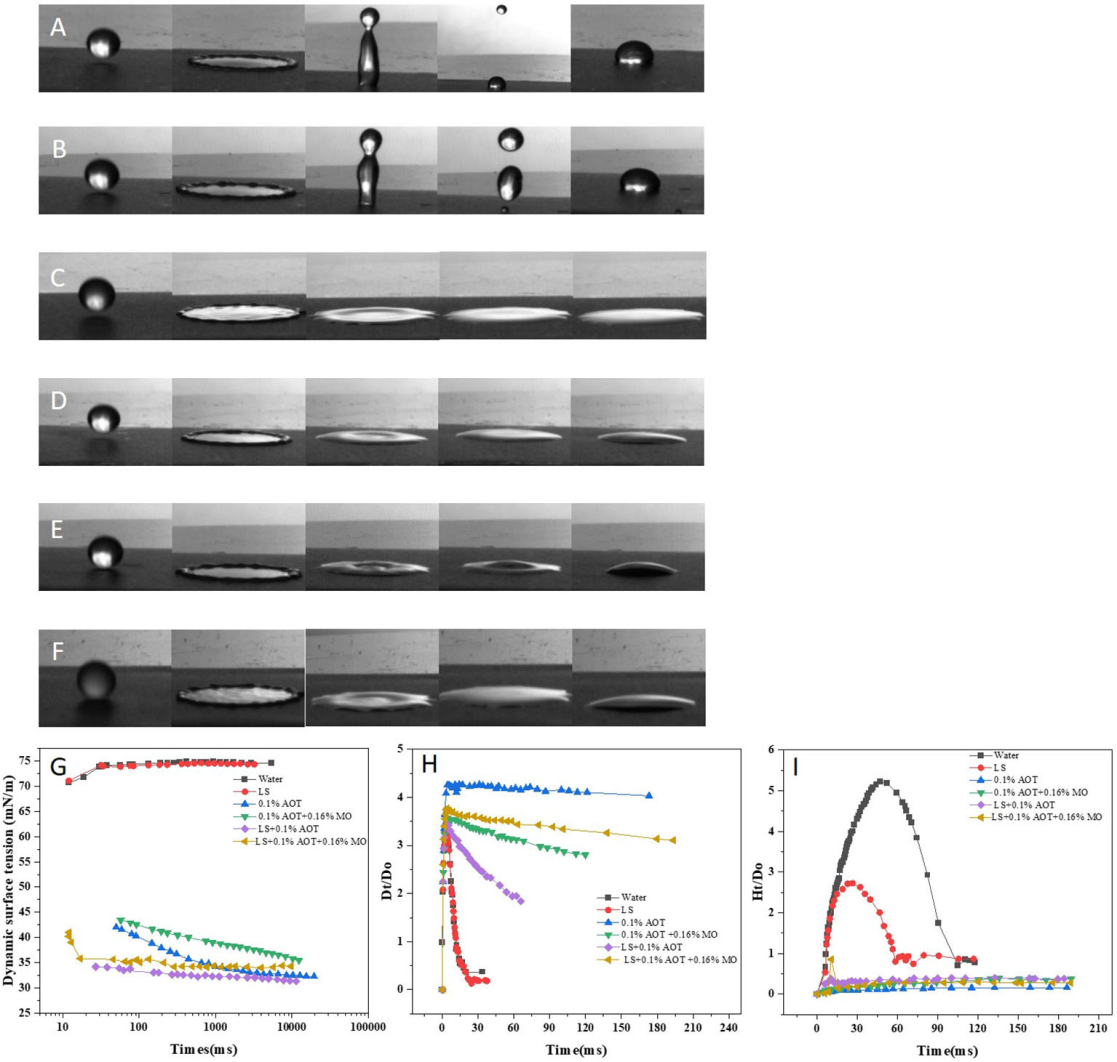
Impact process of droplets on a flat paraffin plate. (**A–F**) Impact behaviors of different droplets on a flat paraffin plate. **(A)** Water, **(B)** 0.75 B° LS, **(C)** 0.1% AOT, **(D)** 0.1% AOT + 0.16% MO, **(E)** 0.75 B° LS + 0.1% AOT, and **(F)** 0.75 B° LS + 0.1% AOT + 0.16% MO. **(G)** Dynamic surface tension of water, LS, AOT, AOT + MO, LS + AOT, and LS + AOT + MO. **(H)** Temporal variations in the spreading factors during post-impact spreading on a flat paraffin plate. The treatments were water, 0.75 B° LS, 0.1% AOT, 0.1% AOT + 0.16% MO, 0.75 B° LS + 0.1% AOT, 0.75 B° LS + 0.1% AOT + 0.16% MO. **(I)** Temporal variations in the bounce factor during post-impact spreading on a flat paraffin plate. The treatments were water, 0.75 B° LS, 0.1% AOT, 01% AOT + 0.16% MO, 0.75 B° LS + 0.1% AOT, 0.75 B° LS + 0.1% AOT + 0.16% MO. AOT: dioctyl sulfosuccinate sodium salt MO: methyl oleate LS: lime sulfur B°: baume degrees.

In the field, the effective deposition of droplets on the target interface is key to improving the efficacy of pesticides. We compared the effect of droplets of different solutions on the surface of flat paraffin plates, revealing the dynamics of droplets when they collided with the paraffin surface, spread out, and subsequently rebounded. The impact velocity of the droplet was 2 m/s. Considering the influence of adjuvants on the droplet size, we used needles of two specifications (GB/T 1962.1-2015) to ensure that the droplet size was 2 ± 0.2 mm (**Figure 3**). When the droplets of water or LS solutions (**Figures 3A, B**) fell on the surface of the flat paraffin plate, they first spread out after contacting the surface of the plate followed by a high bounce. During the bounce process, the droplets broke and finally fell on the surface of the plate in a Cassie-Baxter state. When the droplets of adjuvants or adjuvants and LS (**Figures 3C–F**) fell on the surface of the flat paraffin plate, the diffusion phenomenon occurred first. Unlike droplets of water or LS, those of adjuvants did not bounce, but diffused on the surface of the flat paraffin plate in different states; the combination of 0.1% AOT and LS + 0.1% AOT + 0.16% MO had a larger diffusion area than water and LS.

Further, we measured the dynamic surface tension (**Figure 3G**) of the solutions over time and calculated the change in spreading factor D_t_/D_0_ (**Figure 3H**) and bounce factor H_t_/D_0_ (**Figure 3I**) with time, during post-impact spreading on the flat paraffin plate. Dynamic surface tension affects droplet behavior at the target interface, and the surfactant with low dynamic surface tension is more helpful in inhibiting droplet rebound on superhydrophobic surfaces. The dynamic surface tension between water and LS was 70-75 mN/m (**Figure 3G**). However, the dynamic surface tension of LS with adjuvants was considerably reduced to 30-40 mN/m, which could reduce or inhibit the splash bounce of pesticide droplets at the target interface; this was consistent with the results of high-speed photography.

The curve of the spreading factor D_t_/D_0_ and bounce factor H_t_/D_0_ over time revealed the impact behavior of the droplets at the target interface. These help accurately describe the spreading and bouncing behavior of the droplet at the target interface in detail. The diameter of the nearly spherical droplet before hitting the target was D_0_, and the droplet spread out after contacting the target interface. All the droplets spread out to a maximum area within 3 ms, but the droplet spreading factors were different. Water and LS droplets rapidly shrank back after spreading to the maximum area, and the spreading factor was less than 0.5 on the target surface within 30 ms. When water or LS droplets with adjuvants were spread to the largest area, the droplets of LS + 0.1% AOT tended to shrink; however, the droplets of 0.1% AOT and LS + 0.1% AOT + 0.16% MO maintained a large diffusion area, which was conducive to further absorption, penetration, and conduction of droplets. The diameter of the nearly spherical droplet before hitting the target was D_0,_ the droplet maybe bounces in different degrees after touching the target interface. Droplets of water and LS bounce when they touch the interface, the bounce factor of water and LS are 5.21 and 2.72 respectively, however, the droplets added with adjuvants did not exhibit bouncing behavior at the target interface.

### 3.5 Impact behavior of a droplet on a petal surface

**Figure 4:**
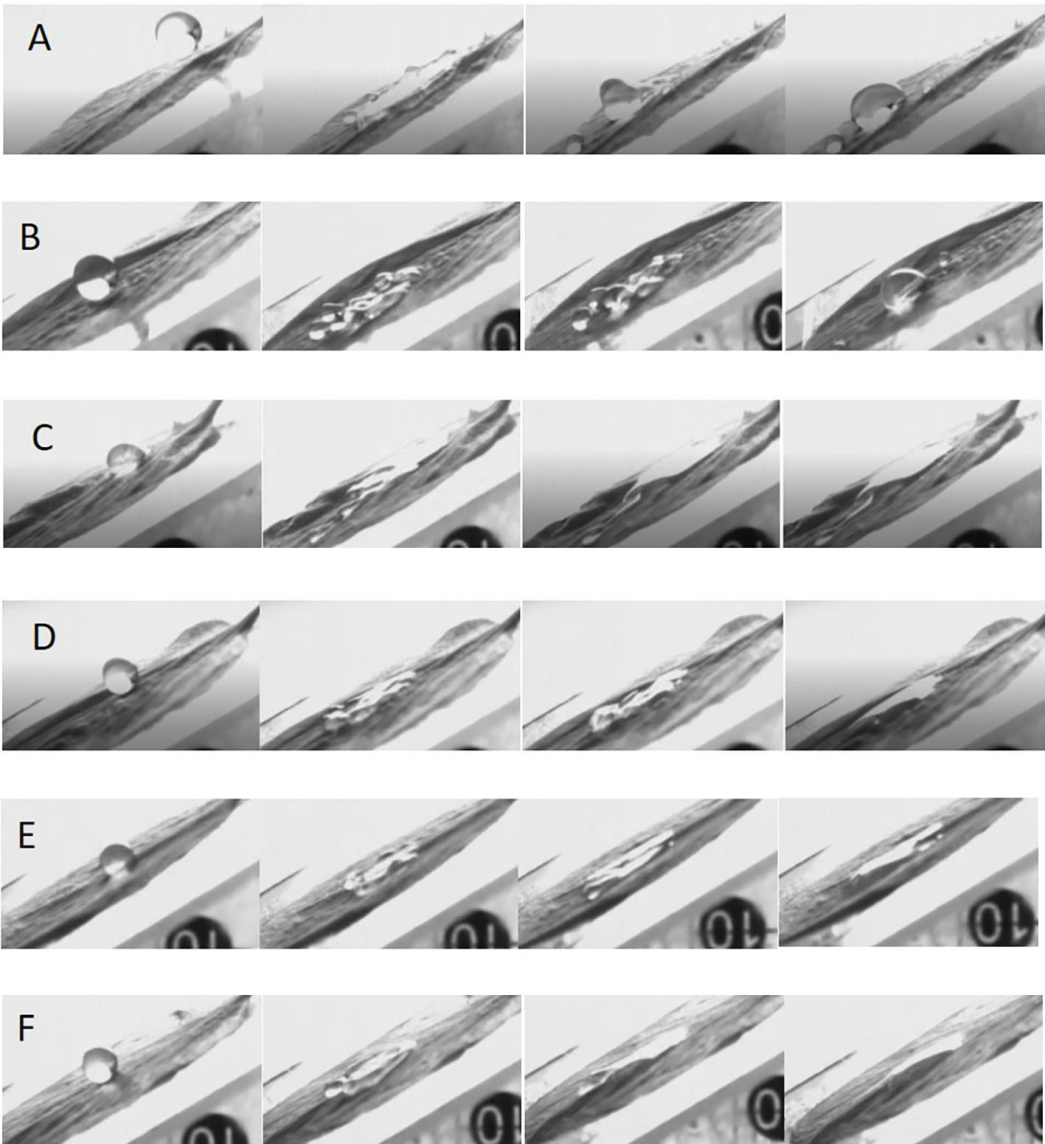
Impact process of droplets on the petal surfaces. **(A–F)** Impact behaviors of different droplets on petal surface. **(A)** water, **(B)** 0.75 B° LS, **(C)** 0.1% AOT, **(D)** 0.1% AOT + 0.16% MO, **(E)** 0.75 B° LS + 0.1% AOT, and **(F)** 0.75 B° LS + 0.1% AOT + 0.16% MO. AOT, dioctyl sulfosuccinate sodium salt; MO, methyl oleate; LS, lime sulfur; B°, baume degrees.

Since the leaves and flowers apparatus of most plants are inclined, it is necessary to study the effect of droplets on inclined hydrophobic surfaces. Apple petals with a tilt angle of 30° were used as the hydrophobic surface to measure the impact behavior of droplets on the target interface (**Figure 4**). At 30°, water and pesticide droplets broke up into smaller droplets and slid off, settling on the petal surface in a Cassie-Baxter state (**Figures 4A, B**). After the addition of adjuvants to water or LS, the droplet deposition state on the petals significantly improved. The droplets deposited in a larger area on the petal surface, close to the Wenzel state; this was conducive to the deposition of flower thinning agent on the surface of petals and promoted the absorption, penetration, and conduction of pesticide and further improved the efficacy of flower thinning.

### 3.6 The spreading behavior of a droplet on pistils and stamens

**Figure 5:**
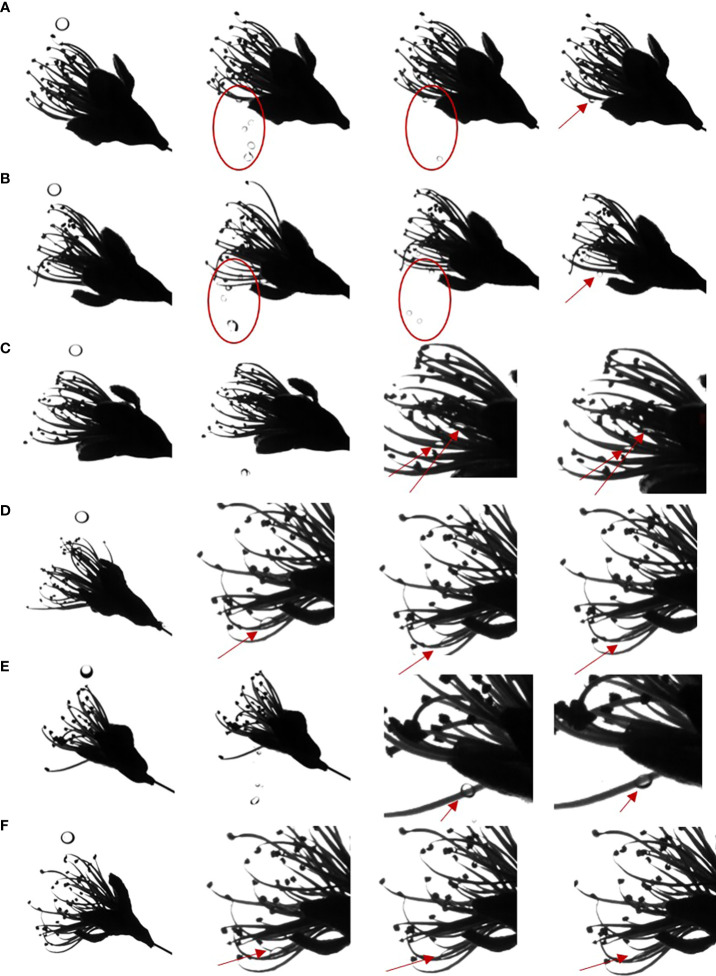
Spreading process of droplets on the pistil and stamen. **(A–F)** The spreading behavior of droplets on the pistil and stamen. **(A)** Water, **(B)** 0.75 B° LS, **(C)** 0.1% AOT, **(D)** 0.1% AOT + 0.16% MO, **(E)** 0.75 B° LS + 0.1% AOT, and **(F)** 0.75 B° LS + 0.1% AOT + 0.16% MO. AOT, dioctyl sulfosuccinate sodium salt; MO, methyl oleate; LS, lime sulfur; B°, baume degrees.

The mechanism of action of a flower thinning agent is to burn the flower organs and hinder pollination and fertilization processes so that fruits cannot be fertilized, and fall off from the tree body. Therefore, we studied the spreading behavior of droplets on the flower organs, pistils and stamens. We used droplets with the same diameter and impact velocity. When water or LS droplets impacted the pistil or stamen, the droplets would hang on the pistil or stamen as shown by the red circle and arrow in **Figures 5A, B**, and the droplets hung in a spherical shape for a sustained period of time. When the adjuvant droplet impinged on the stamen or pistil, the droplet hung on the stamen. However, after a while, the droplet spread on the stamen and finally deposited in a large area on the stamen, as shown by the arrow in **Figures 5C–F**. The dynamic impact process of droplets on pistils and stamens can be seen in **Movies S7-S12**. The spreading effect of LS + 0.1% AOT droplets on the stamen was not as good as that of LS + 0.1% AOT + 0.16% MO droplets. This may be because the behavior of droplets on curved surfaces is different from that on plane surfaces, this process requires both good spreading and infiltration effects.

Because of the small contact area between stamens and pistils, surface properties, such as contact angle, cannot be measured. By measuring the impact behavior of droplets, the spreading behavior of droplets on this curved target surface can clearly be seen. The effect of the flower thinning agent provides a theoretical guidance for the study of the behavior of the adjuvants on curved surfaces.

### 3.7 The permeability and spreading ability of the droplets using the fluorescence tracer method

**Figure 6:**
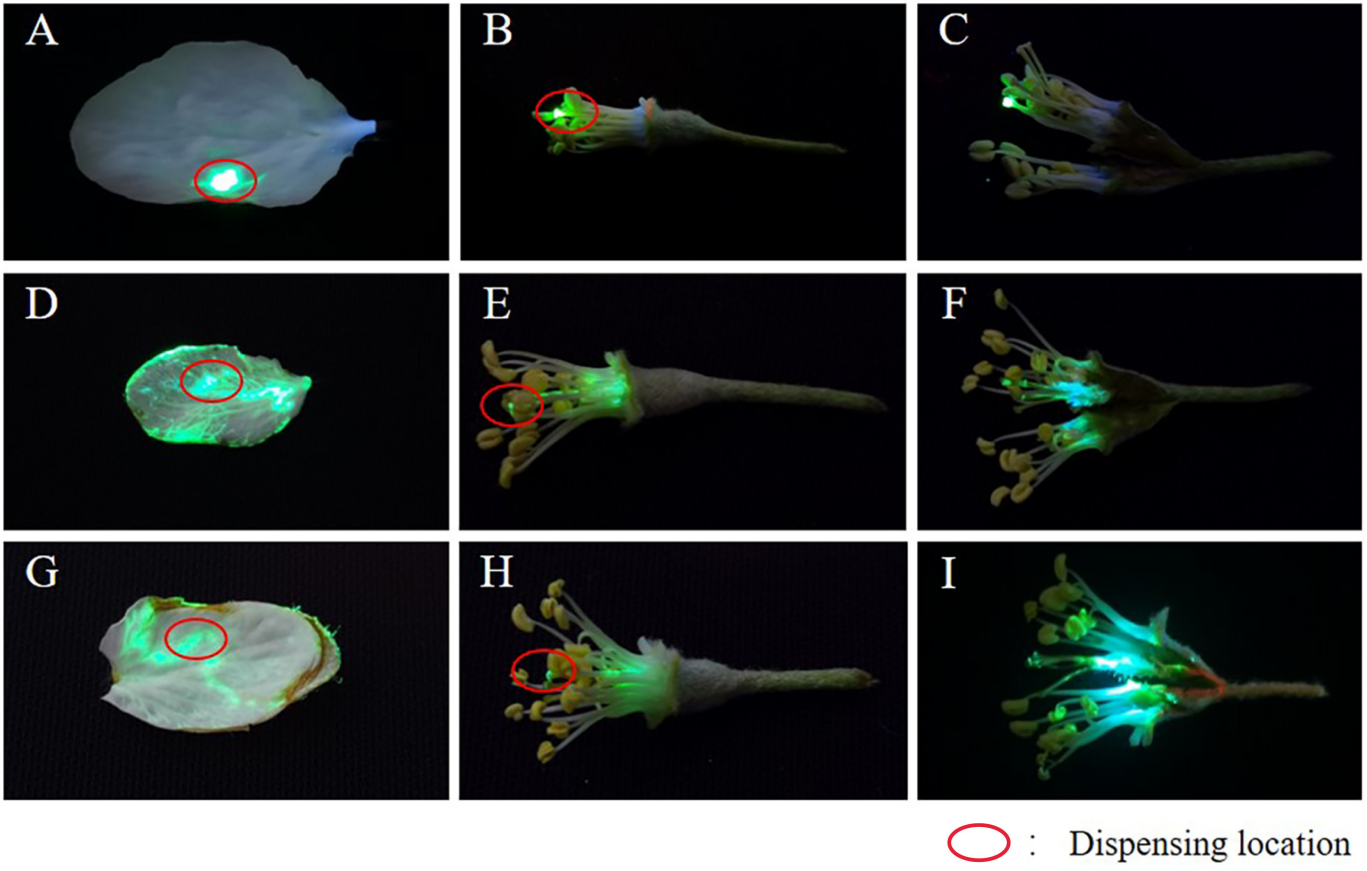
Permeability and spreadability behavior of the droplets estimated using the fluorescent tracer method. (**A–I**) The permeability and spreadability behavior of the droplets using the fluorescent tracer method. **(A-C)** pyranine, **(D-F)** pyranine + 0.1% AOT, and **(G-I)** pyranine + 0.1% AOT + 0.16% MO. AOT, dioctyl sulfosuccinate sodium salt; MO, methyl oleate.

To assess the spreading and penetration effect of adjuvants more directly, we used the fluorescent tracer method. The fluorescent dye chosen was pyranine, which exhibits green fluorescence at 365 nm under ultraviolet illumination. The platform for fluorescence tracing is shown in **Figure S1**. A dark environment is required during photography to ensure that the fluorescence color can be captured clearly and accurately. During the experiment, we first applied pyranine with or without adjuvants on naturally growing petals and stamens, as shown by the red circle on the petals and stamens in **Figure 6**. To avoid the influence of sunlight, rain, and dew on the test results, the petals and stamens were removed for indoor testing after 12 h of application. No spreading and permeating behavior occurred on petals and stamens after spot coating of pyranine, and fluorescence remained unchanged at the spot coating position (**Figures 6A–C**). In contrast, such behaviors did occur after the application of pyranine with adjuvants [**Figures 6D–F** (pyranine + AOT) and **Figures 6G-I** (pyranine + AOT + MO)].

In particular, after application of the pyranine + AOT + MO combination, the droplet deposition on the curved target increased. This indicated that owing to the special structure of the curved target, adjuvants need to be added to liquids to increase permeability and spread ability to promote absorption of the liquid. This can reduce the loss of pesticides, and improve its use rate.

### 3.8 Field experiments

#### 3.8.1 Effects of various thinning treatments on flowers

Experimental information on the effects of different treatments on flowers is summarized in support Information (SI).

Spraying water had no effect on flower growth. Spraying 0.1% AOT, 0.165% MO, and 0.03% emulsifier negatively affected the growth of petals but had no effect on the growth of stamens and pistils. Spraying various concentrations of LS or LS + 0.1% AOT + 0.16% MO significantly negatively affected the growth of flowers. The 0.75 B° + 0.1% AOT + 0.16% MO solution was selected for further study as it provided optimum experimental results.

#### 3.8.2 Analysis of parameters of apple thinning

**Figure 7:**
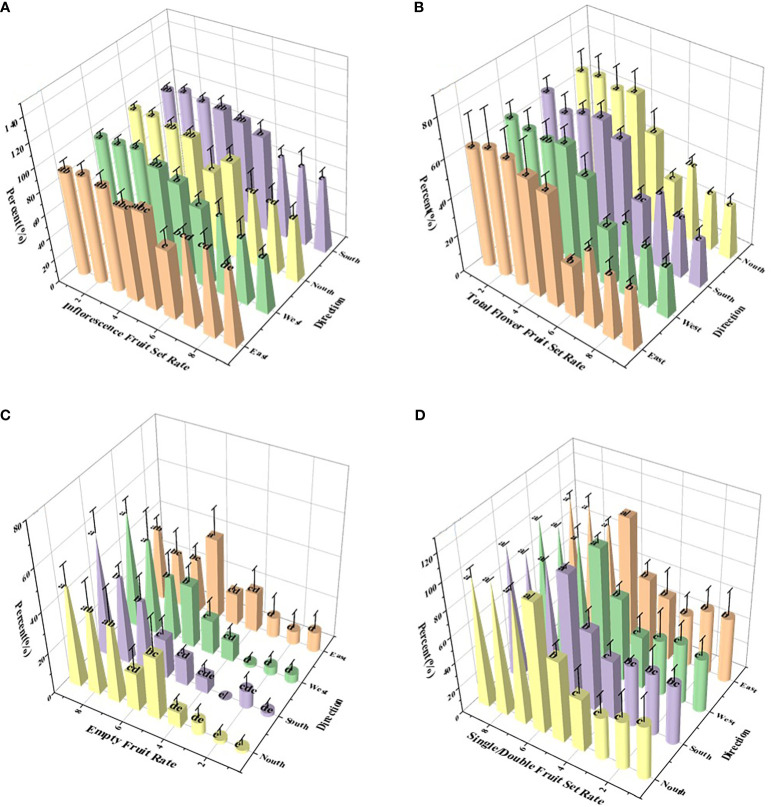
Effect of different treatments on apple thinning. **(A)** Inflorescence fruit setting rate; **(B)** total flower fruit setting rate; **(C)** single/double fruit setting rate; and **(D)** empty fruit rate. On the abscissa, treatments 1 - 9 are water, 0.1% AOT, 0.1% AOT + 0.16% MO, 0.5 B° LS, 0.75 B° LS, 1 B° LS, 0.75 B° LS + 0.1% AOT, 0.75 B° LS + 0.1% AOT + 0.16% MO, and artificial thinning, respectively. AOT, dioctyl sulfosuccinate sodium salt; MO, methyl oleate; LS, lime sulfur; B°, baume degrees.

The effect of adjuvants on the spreading, wetting, and penetration behavior of droplets on petal and stamen surfaces has been established. Based on the results, field experiments on flower thinning were performed to further evaluate the effect of LS with or without adjuvants on thinning. Pesticide spraying was performed twice in the flowering stage, and statistical analysis of the field data was performed after the apples were set (**Figure 7**).

Various levels of LS exhibited significantly different flower thinning effects on apples, but the effects were better than the water control. At 0.5, 0.75, and 1 B° LS, the inflorescence fruit setting rates were 88.73%, 81.87%, and 74.93%, respectively (**Figure 7A**), which were higher than those by artificial thinning. However, the inflorescence fruit setting rate of LS and adjuvants combination was similar to that of artificial thinning (52.51%), and that of 0.1% AOT + 0.16% MO (62%) was better than that of 0.1% AOT (68.38%). Further, we sprayed adjuvants alone (treatments 2 and 3 in the **Figure 7**), and the inflorescence fruit setting rate was comparable to that of water. The empty fruit rate exhibited the same trend as that of the inflorescence fruit set rate (**Figure 7D**).

At 0.5, 0.75, and 1 B° LS, the total flower fruit setting rate was 63.75%, 54.65%, and 30.42%, respectively, whereas that of artificial thinning was 27.33%. The total flower fruit set rate of the LS and adjuvants combinations was 38.64 (treatment 7) and 30.87 (treatment 8). Further, after spraying the adjuvant alone (treatments 2 and 3 in the **Figure 7**), the total flower fruit setting rate was over 60%. The differences in single fruit set rate and flower thinning rate among various treatments can be seen in **Table S1**. The total flower fruit setting rate is closely related to the single and double fruit set rates, and the single and double fruit rates positively affect the quality of the fruit. In **Figure 7C**, we can see that the single and double fruit set rates of treatments 6, 7, 8, and 9 were close to 100%. However, as mentioned before, the inflorescence fruit set rate of treatment 6 was too high, and that of treatments 7 and 8 was close to that of artificial thinning.

#### 3.8.3 Determination of fruit quality and economic valuation

After analyzing the data of the thinning test, an artificial thinning treatment was conducted for some treatments (except water treatment) to avoid overhanging fruits and detrimental effects on fruit growth. Fruit that has not been thinned loses approximately 20% of its weight, which affects not only the quality of the fruit but also the healthy growth of the tree. For Yueguan apples, the addition of flower thinning agent and adjuvants had no adverse effect on the sensory quality of apples, which is an important consideration while using flower thinning agents (**Table 3**).

**Table 3:** Effect of various thinning treatments on fruit quality.

Treatment	Fruit Mass (g)	Soluble Solids (%)	Fruit Firmness (Kg/cm^2^)	Fruit Shape Index
Water	103.46 ± 6.80b	12.09 ± 0.91ab	10.83 ± 0.84bc	0.85 ± 0.05a
0.1%AOT	108.32 ± 3.41b	11.67 ± 0.86b	11.04 ± 0.55ab	0.87 ± 0.07a
0.1% AOT + 0.16% MO	108.45 ± 6.59b	12.00 ± 0.80ab	11.06 ± 0.49ab	0.86 ± 0.06a
0.5 B° LS	126.94 ± 5.52a	12.15 ± 0.95ab	10.38 ± 0.46c	0.86 ± 0.03a
0.75 B° LS	126.35 ± 7.62a	12.11 ± 0.87ab	10.77 ± 0.95bc	0.88 ± 0.05a
1 B° LS	129.42 ± 5.93a	12.11 ± 0.76ab	10.91 ± 0.49bc	0.88 ± 0.05 a
0.75 B° LS +0.1%AOT	129.01 ± 8.52a	12.33 ± 0.61ab	11.57 ± 0.51a	0.87 ± 0.08a
0.75 B° LS + 0.1% AOT + 0.16% MO	127.80 ± 4.71a	12.56 ± 0.52a	11.13 ± 0.53ab	0.89 ± 0.05a
Artificial	130.82 ± 4.96a	12.03 ± 0.8ab	11.25 ± 0.51ab	0.84 ± 0.05a

The data in the table is the average value of 20 repeated treatments shown in materials and methods, and the data in the same column marked with different lowercase letters indicate significant differences (p<0.05).

AOT, dioctyl sulfosuccinate sodium salt; MO, methyl oleate; LS, lime sulfur; B°, baume degrees.

The variety of apple is “Yue Guan”.

At the same time, we calculated the input cost difference between a chemical flower thinning agent and artificial thinning (**Table 4**). The experimental values of the thinning agent were taken as the standard, and costs calculated into hectares. The cost of chemical flower thinning was only 20% of that of the labor cost for manual thinning. Moreover, the combination of chemical flower thinning agent and adjuvants not only achieved better effects than higher concentrations of thinning agent alone, but also costed less and had a higher usage value.

**Table 4:** Average dosage and cost comparison chart of various thinning treatments.

Treatment	Dosage (kg/ha^2^)	Cost (yuan/ha^2^)	Account for the Percentage of Artificial (%)
0.5 B° LS	7.50	367.5 + 450	22.71
0.75 B° LS	8.55	418.95 + 450	24.14
1 B° LS	10.00	490.25 + 450	26.12
0.75 B° LS +0.1%AOT	7.5 + 0.015	418.95 + 15 + 450	24.55
0.75 B° LS + 0.1% AOT + 0.16% MO	7.5 + 0.045	418.95 + 18 + 450	24.64
Artificial		3600.00	100.00

AOT, dioctyl sulfosuccinate sodium salt; MO, methyl oleate; LS, lime sulfur; B°, baume degrees.

The variety of apple is “Yue Guan”.

## 4 Conclusion

In summary, this study systematically explored the synergistic effects of addition of adjuvant AOT and (AOT +MO) into LS in the chemical desensitization process from three perspectives: indoor physical and chemical properties, nature of the physical target, and field experiments. The addition of adjuvants could effectively reduce the static surface tension from 72mN/m to 28mN/m and 32mN/m respectively, increase the spreading factor from 0.18 to 1.83 and 3.10 respectively, reduce the bounce factor from 2.72 to 0.37 and 0.27 respectively, and increase the deposition amount of the droplets on target interface. In particular, the increase in solution spread ability and permeability caused by adjuvants increased droplet deposition in the flower and paraffin surfaces, such as stamens, and promoted the absorption. The field test results revealed that the flower thinning rate of adjuvant and non-adjuvant were 80.55% and 54.4% respectively, and the flower thinning effect of adding adjuvant was the same as that of artificial which the flower thinning rate was 84.77%. The quality of apples treated with adjuvants was similar to that treated with artificial, and the weight of single fruit increased by 24.08% compared with CK (spray water). In this study, AOT and MO were used as adjuvants to improve the efficiency of flower thinning agents. Most of the current studies focus on the effect of adjuvants on pesticide, but there are seldom related studies on the effect of additives on plant growth regulators. This work not only provided guidance for increasing the deposition and spreading of droplets on the hydrophobic interface of petals, stamens and leaves, but also expanded the application of adjuvants in plant growth regulators, and promoted the sustainable green development of agriculture.

## Author's note

Due to the high seasonal requirements of test materials and the small volume stamens and pistils of apples, the stamens and pistils used in the test were peach blossoms belonging to the Rosaceae family.

## Data availability statement

The raw data supporting the conclusions of this article will be made available by the authors, without undue reservation.

## Author contributions

ZZ: Conceptualization, Funding acquisition, Project administration, Resources, Supervision, Validation, Writing – review & editing. YYL: Data curation, Formal Analysis, Investigation, Methodology, Software, Writing – original draft, Writing – review & editing. YL: Data curation, Investigation, Writing – review & editing. CW: Data curation, Writing – review & editing. RZ: Data curation, Software, Writing – review & editing. ML: Investigation, Writing – review & editing. JC: Methodology, Writing – review & editing. LM: Methodology, Writing – review & editing. XH: Conceptualization, Supervision, Writing – review & editing. XW: Conceptualization, Supervision, Writing – review & editing.
