# Supplementary material for: Effect of adjuvants on physicochemical properties of lime sulfur on flower/paraffin and application on flower thinning
Source: Front Plant Sci. 2023 Sep 11;14:1257672. doi: 10.3389/fpls.2023.1257672 (PMC10536241; doi:10.3389/fpls.2023.1257672)
Supplement: Supplementary file 2 [file DataSheet_2.docx]

**Supporting Information**

**Movie S1.** The impact process of water on paraffin surfaces.

**Movie S2.** The impact process of LS (0.75° Be) on paraffin surfaces.

**Movie S3.** The impact process of 0.1% AOT on paraffin surfaces.

**Movie S4.** The impact process of 0.1% AOT + 0.16% MO on paraffin surfaces.

**Movie S5.** The impact process of LS (0.75° Be) + 0.1% AOT on paraffin surfaces.

**Movie S6.** The impact process of LS (0.75° Be) + 0.1% AOT + 0.16% MO on paraffin surfaces.

The droplets of 0.1% AOT had a perfect spreading property on the surface of paraffin surface and had the largest spreading area. However, the droplets of LS + 0.1% AOT had a large retraction process after spreading on the surface of paraffin surface, making the final spreading area of droplets on the surface of paraffin smaller. The results show that the compatibility between LS and AOT adjuvants are not good.

**Movie S7.** The impact process of water on pistils and stamens.

**Movie S8.** The impact process of LS (0.75° Be) on pistils and stamens.

**Movie S9.** The impact process of 0.1% AOT on pistils and stamens.

**Movie S10.** The impact process of 0.1% AOT + 0.16% MO on pistils and stamens.

**Movie S11.** The impact process of LS (0.75° Be) + 0.1% AOT on pistils and stamens.

**Movie S12.** The impact process of LS (0.75° Be) + 0.1% AOT + 0.16% MO on pistils and stamens.

The video shows the spreading and penetration behavior of different droplets on pistils and stamens. The droplets of water and LS finally appear as larger droplets on pistils and stamens. The droplets added with AOT+MO adjuvants have better spreading effect on pistils and stamens. Video 12 shows the impact behavior of the LS+AOT+MO droplet on pistils and stamens. The video was shot at 4021fps. The video shows the behavior of the droplet 1.6s after impact the pistils and stamens, and the droplet finally spreads on the stamen. Because of the adjuvant of the penetration enhancer, the spreading and penetration of the droplets on the stamens are promoted.

**Fluorescent tracer method platform**

**
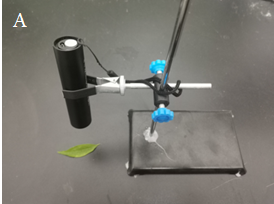

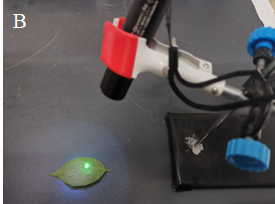
**

**Figure S1.** Fluorescent tracer method platform.

The platform of fluorescence tracing technology is shown in Figure S1 A. After the leaf are applied on fluorescence, it will emit green fluorescence under the irradiation of 365nm ultraviolet lamp (Figure S1 B).

**Effects of various thinning treatments on flowers**

The effect of various thinning treatments on flower behavior was assessed before the thinning experiments. We sprayed the same amount of water, 0.1% AOT, 0.165% MO, 0.03% emulsifier, 0.5 B° LS, 0.75 B° LS, 1 B° LS, 0.5 B° LS + 0.1% AOT + 0.16% MO, 0.75 B° LS + 0.1% AOT + 0.16% MO, and 1 B° LS + 0.1% AOT + 0.16% MO solutions on various branches. Flowers were photographed 3 days after spraying to compare the effects of various thinning treatments on flowers (Figs. S2–S4).


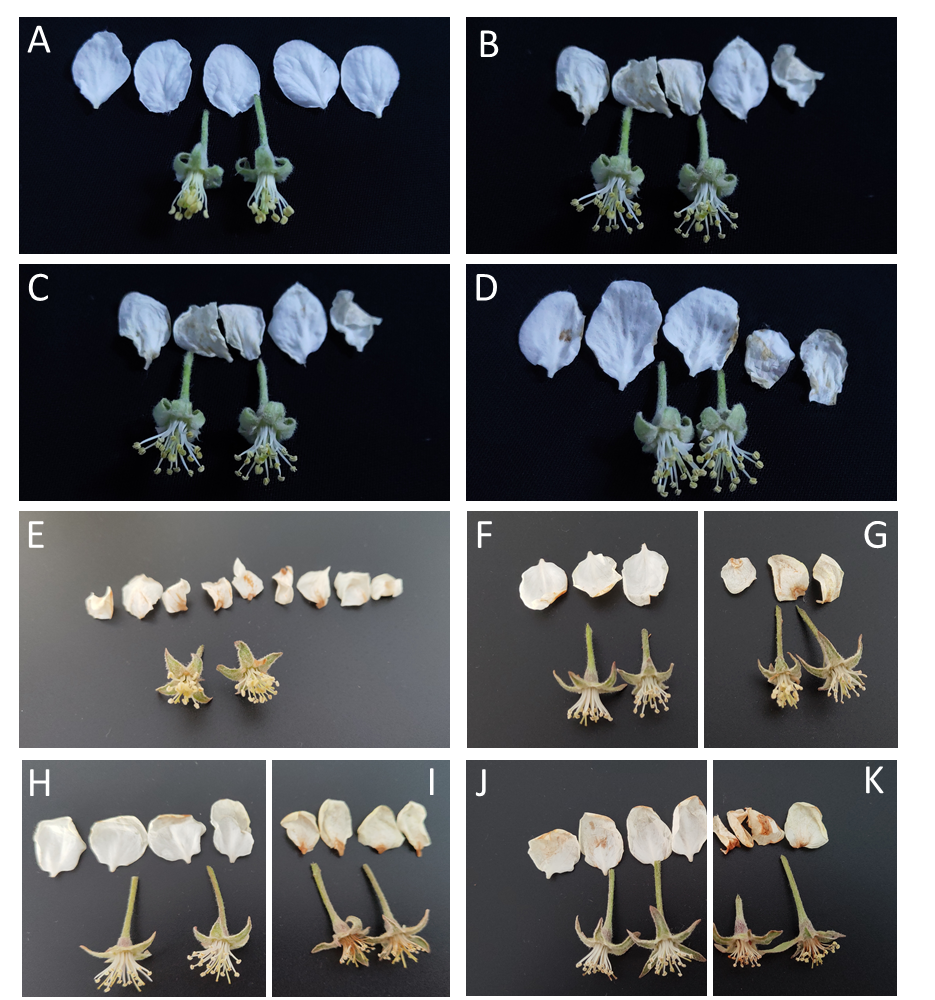


**Figure S2.** Flower effect after spraying different treatment solutions for one day.

A: Water, B: 0.1% AOT, C: 0.03% emulsifier, D: 0.16% MO, E: 0.1% AOT + 0.16% MO, F: 0.5 ° Be LS, G: 0.5 ° Be LS + 0.1% AOT + 0.16% MO, H: 0.75 ° Be LS, I: 0.75 ° Be LS + 0.1% AOT + 0.16% MO, J: 1° Be LS, K:1° Be LS + 0.1% AOT + 0.16% MO.


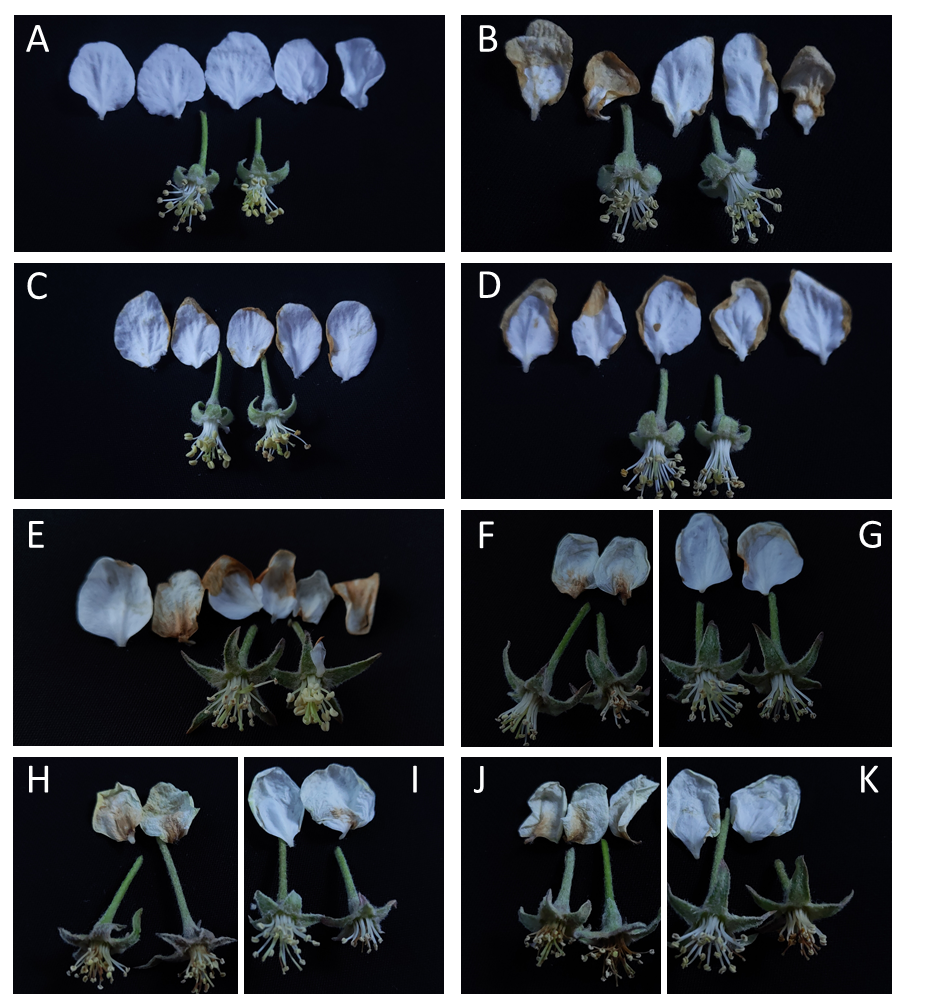


**Figure S3.** Flower effect after spraying different treatment solutions for two days.

A: Water, B: 0.1% AOT, C: 0.03% emulsifier, D: 0.16% MO, E: 0.1% AOT + 0.16% MO, F: 0.5 ° Be LS, G: 0.5 ° Be LS + 0.1% AOT + 0.16% MO, H: 0.75 ° Be LS, I: 0.75 ° Be LS + 0.1% AOT + 0.16% MO, J: 1° Be LS, K:1° Be LS + 0.1% AOT + 0.16% MO.


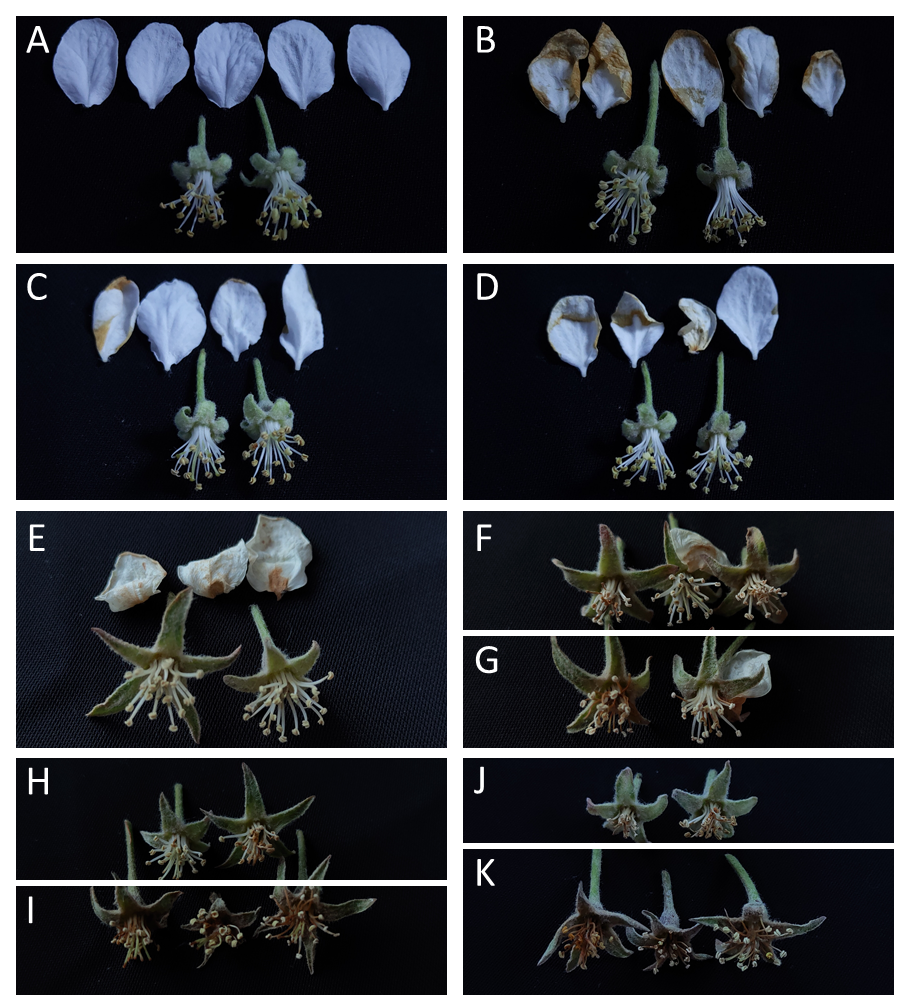


Figure S4. Flower effect after spraying different treatment solutions for three days.

A: Water, B: 0.1% AOT, C: 0.03% emulsifier, D: 0.16% MO, E: 0.1% AOT + 0.16% MO, F: 0.5 ° Be LS, G: 0.5 ° Be LS + 0.1% AOT + 0.16% MO, H: 0.75 ° Be LS, I: 0.75 ° Be LS + 0.1% AOT + 0.16% MO, J: 1° Be LS, K:1° Be LS + 0.1% AOT + 0.16% MO.

When the petals or stamens and pistils turn brown, it is a sign that the spray is affecting their growth and thus thinning the flowers. Spray water will not affect flower growth, while treatments B, C and D will affect petals growth, but this will not affect fruit setting. The Browning degree of petals and stamens deepened with the increase of the spraying concentration. In thinning agent adding adjuvants, degree of Browning of petals and stamens are deepening. From the results of three days, 0.75 ° Be LS + 0.1% AOT + 0.16% MO and 1 ° Be LS + 0.1% AOT + 0.16% MO had similar effects on flower Browning. 0.75 ° Be LS + 0.1% AOT + 0.16% MO was selected as the combined treatment of pesticide and adjuvant for field test. The treatment ensured the efficacy based on reducing the dosage.

Table S1. Effect of different control on apple thinning.

| Treatment | Single Fruit Set Rate (%) | Flower Thinning Rate (%) |
| --- | --- | --- |
| Water | 22.05c | 38.89e |
| 0.1% AOT | 21.56c | 39.48e |
| 0.1% AOT + 0.16% MO | 20.41c | 39.75e |
| 0.5 B° LS | 21.89c | 43.24e |
| 0.75 B° LS | 29.46c | 55.56d |
| 1 B° LS | 70.52a | 76.83bc |
| 0.75 B° LS + 0.1%AOT | 46.52b | 73.87c |
| 0.75 B° LS  + 0.1% AOT + 0.16% MO | 68.84a | 80.55ab |
| Artificial | 75.55a | 84.77a |
